# Digital transformation and the choice of management control modes in enterprise groups

**DOI:** 10.1371/journal.pone.0320328

**Published:** 2025-04-22

**Authors:** Junhui Li, Xianzhi Zhang

**Affiliations:** School of Accounting, Dongbei University of Finance and Economics, Dalian, Liaoning, China; Tianjin University, CHINA

## Abstract

Digital transformation has a significant impact on the choice of management control modes within enterprise groups. This study uses data from publicly listed companies in China from 2010 to 2022 to empirically examine the effect of digital transformation on the management control modes of enterprise groups. It further explores the mechanism and moderating effects of digital transformation in influencing the selection of management control modes. The findings indicate that, under the impact of digital transformation, enterprise groups are more inclined to adopt decentralized management control modes. The mechanism analysis suggests that digital transformation can mitigate principal-agent problems between parent and subsidiary companies by improving internal control quality, thus promoting a decentralized management control mode. The moderating effects reveal that the facilitative impact of digital transformation on choosing a decentralized control mode is more pronounced in state-owned enterprise groups and those operating in environments with higher uncertainty. Moreover, as the level of digital transformation or corporate governance improves, enterprise groups are more likely to adopt decentralized management control modes. This study extends the measurement indicators for the choice of management control modes in enterprise groups across four dimensions: personnel authority, operational authority, investment authority, and financial authority, and constructs a research framework to reveal the practical effects of China’s digital transformation strategy.

## 1. Introduction

Management control is a crucial tool for enterprises to achieve their strategic goals, and the choice of management control modes is key to achieving high-quality development (Zhang et al., 2017) [1]. Group and diversified structures have become the main characteristics of Chinese enterprises (Liu et al., 2024) [2]. As the uncertainty of the business environment increases, traditional management control modes face new challenges. For example, many enterprise groups select management control modes with incomplete scopes and insufficient control efforts, which prevents them from making effective business decisions. In the digital economy era, digital transformation provides new methods for management control in enterprise groups. Through digital technology integration, data sharing, departmental coordination, refined control, and real-time monitoring, enterprise groups could address weak control foundations and low control efficiency (Yuan et al., 2021; Wu et al., 2021; Li et al., 2022) [3–5]. Digital transformation has gradually become an important means for improving the efficiency of management control in enterprise groups.

In recent years, the topic of digital transformation has gained growing attention in academia. Scholars increasingly recognize that emerging technologies such as blockchain, artificial intelligence, and cloud computing are reshaping business models (Austin and Williams, 2021) [6]. During digital transformation, digital technologies are applied to various domains, including risk management and decision-making, data-driven knowledge, and information communication (Urbinati et al., 2019; Lin et al., 2024) [7,8]. Existing research explores the economic consequences of digital transformation from diverse perspectives, such as corporate investment and financing activities (Lin et al., 2024; Kim et al., 2018) [8,9], output and performance (Barba Sanchez et al., 2018; Fang and Liu, 2024) [10,11], capital market performance and firm value (Wu et al., 2022; Zhao and Fang, 2023) [12,13], and environmental performance (Singh et al., 2021; Wang et al., 2023; Zhou and Liu, 2023) [14–16]. However, few studies extend the exploration of the economic consequences of digital transformation to the internal dynamics of enterprise groups, specifically the management control modes between parent and subsidiary companies.

The essence of choosing management control modes for enterprise groups is the allocation of power between the parent and subsidiary companies (Chen, 2007) [17]. Centralized control refers to a mode where decision-making power, operating authority, investment rights, and financial control are concentrated in the parent company, while decentralized control distributes these powers to the subsidiaries (Pan et al., 2018) [18]. Jensen and Meckling (1992) pointed out that the effective allocation of power at different levels of an enterprise should minimize the sum of knowledge transfer costs and agency costs [19]. More specifically, if a subsidiary has a significant amount of proprietary knowledge, a decentralized management control mode can combine authority with this knowledge, minimizing knowledge transfer costs. In contrast, a centralized mode increases these costs. In a decentralized management control mode, the misalignment of goals between the parent and subsidiary companies causes agency costs, which rise with the degree of decentralization (Zhang et al., 2017) [1]. Existing literature explains the mechanism for choosing management control modes by finding the optimal position for power that minimizes the total costs associated with knowledge transfer and agency. In other words, the choice between centralized and decentralized management control modes depends on balancing knowledge transfer and agency costs (Jensen and Meckling, 1992) [19].

In the digital economy era, digital transformation reshapes business models, operational management, and business processes across the board (Qi et al., 2021; Shen et al., 2022) [20,21]. By lowering both knowledge transfer and agency costs between parent and subsidiary companies, digital transformation balances the opposing forces between knowledge transfer and agency costs, thereby influencing the selection of management control modes (Lin et al., 2024) [8]. A critical factor in balancing these costs lies in eliminating information asymmetry during knowledge and information transfer within enterprise groups (Pan et al., 2018) [18]. On the one hand, digital transformation enables the transfer of more accurate and reliable knowledge to parent company management, equipping them with the specialized knowledge required for decision-making and reducing knowledge transfer costs (Li et al., 2022) [5]. On the other hand, digital technologies provide parent companies with transparent oversight platforms, directly reducing agency costs between parent and subsidiary companies (Liu et al., 2024) [2]. Thus, digital transformation facilitates an optimal boundary for power allocation within enterprise groups by balancing knowledge transfer and agency costs. However, as digital transformation deepens, how can enterprise groups make full use of digital technology to choose management control modes? This question remains unanswered.

Existing literature, largely grounded in agency theory (Jensen and Meckling, 1995) [22], investigates the factors influencing management control modes selection from perspectives such as parent-subsidiary characteristics (Negandhi and Reimann, 1973; Dau et al., 2021; Cheng et al., 2024) [23–25], external factors faced by enterprises (Bloom et al., 2014; Popli et al., 2017; Luo et al., 2023) [26–28], and managerial characteristics and internal governance (Hempel et al., 2012; Graham et al., 2015; Firk et al., 2024) [29–31]. Most studies focus on the progression of digital transformation amid waves of technological iteration. However, the “information extraction” and “cost-efficiency improvement” effects driven by digital transformation significantly influence the decision-making resources (information) and management styles within enterprise groups, ultimately affecting their management control modes selection. Therefore, exploring the impact of digital transformation on the factors influencing management control modes selection from this perspective holds substantial theoretical significance.

Based on the analysis above, this study uses data from Chinese listed companies from 2010 to 2022 to empirically examine the impact of digital transformation on the management control modes of enterprise groups. Furthermore, we develop a path analysis framework to analyze how internal and external characteristics of enterprise groups mediate this relationship. The results indicate that digital transformation promotes the adoption of decentralized management control modes in enterprise groups. Mechanism test reveals that this effect is achieved through improvements in internal control quality. Moderating effect tests further show that digital transformation’s promotion of decentralized management control modes is more pronounced in state-owned enterprise groups and those operating under high environmental uncertainty. Additionally, higher levels of digital transformation or corporate governance are associated with a stronger tendency to adopt decentralized management control modes.

The potential contributions of this study are as follows: (1) It expands the metrics for choosing management control modes in enterprise groups. While existing literature often uses single indicators like decision-making authority allocation, this paper develops a comprehensive metric, including dimensions like personnel authority, operational authority, investment authority, and financial authority, synthesizing them into a comprehensive indicator for management control modes. Compared to existing metrics, the measures established in this study better reflect the strategic positioning for high-quality development in enterprise groups. (2) Theoretically, this study incorporates the impact of digital transformation into Jensen and Meckling’s (1995) classical framework for management control [22], analyzing how digital technologies influence knowledge transfer and agency costs. By developing a framework for how digital transformation shapes management control modes selection, this study uncovers the “black box” of how digital transformation drives management control decisions in enterprise groups, expanding the research boundaries between digital transformation and management control modes selection. (3) Practically, this study reveals the implementation effects of China’s digital transformation strategy. As a reform process powered by digital technologies, digital transformation helps enterprise groups improve management control efficiency and value creation (Zhu et al., 2024) [32]. By exploring its impacts on firms at the micro level, this study provides insights and guidance for achieving high-quality development through digital transformation.

## 2. Literature review and hypotheses

### 2.1. Literature review

#### 2.1.1. Digital transformation.

Digital transformation refers to the process by which the integrated application of digital technologies triggers significant changes in the organizational characteristics of enterprises, restructures organizational structures and operational systems, and reshapes value creation methods (Vial, 2019; Sui et al., 2024) [33,34]. Existing literature primarily examines the impact of digital transformation on enterprises from two perspectives: information acquisition and knowledge transfer.

From the information acquisition perspective, studies focus on how digital transformation influences the internal information governance process of enterprises. Pereira et al. (2019) found that the integration of digital technologies eliminates information asymmetry across different modules within an enterprise [35], thereby reducing supervision and governance costs during the management control process. Specifically, the embedding of digital technologies facilitates the digital transformation and upgrade of management control modes, making internal communication more seamless (Wu et al., 2021) [4]. Moreover, while improving the reliability of information and effectiveness of control within management systems typically incurs high costs, the information governance effects brought by digital technologies significantly reduce the interaction costs between internal modules (Sui et al., 2024) [34]. As a result, digital transformation not only enhances information processing capabilities but also alters the traditional information structure of enterprises (Adner et al., 2019) [36], giving rise to decentralized grid-like organizations that favor management control (Xie et al., 2020; Zhu et al., 2023) [37,38].

From the knowledge transfer perspective, the emphasis lies on how knowledge is used within enterprises in the digital era. Digitalization transforms the methods of knowledge transfer within enterprises, enabling large-scale storage of knowledge and intelligent operational modes (Vial, 2019; Zhu et al., 2023) [33,38]. This accelerates knowledge diffusion and sharing, providing decision-makers with a panoramic platform for management control (Bloom et al., 2014; Zorina et al., 2021) [26,39]. In this context, enterprises can use digital technologies to access implicit knowledge in real time, significantly reducing trial-and-error costs associated with utilizing tacit knowledge in different scenarios (Qi and Xiao, 2020) [40]. Consequently, studies have found that digitalization changes the cost of knowledge acquisition within firms, thereby reshaping the conditions for matching power with knowledge, improving oversight efficiency, and elevating digital technologies as a key tool for management control (Brynjolfsson and McElheran, 2016; Goldfarb and Tucker, 2019; Tian and Shi, 2024) [41–43].

#### 2.1.2. Choice of management control modes in enterprise groups.

According to agency theory, centralized management reduces agency costs but results in higher knowledge transfer costs, while decentralized management lowers knowledge transfer costs but leads to increased agency costs (Jensen and Meckling, 1995) [22]. Thus, matching knowledge with authority is key to balancing knowledge transfer and agency costs within enterprise groups and ultimately determining the optimal management control mode (Hayek, 1945) [44]. Based on these findings, scholars have examined management control modes selection from both internal and external perspectives.

From the internal perspective, the focus is on the efficiency of resource management within enterprise groups. Colombo and Delmastro (2004) found that centralized management is necessary for resolving conflicts, balancing interests, maximizing group-wide benefits [45], and preventing subsidiaries’ short-term behavior from undermining the value of parent-subsidiary collaboration (Schuster and Dufek, 2004) [46]. Meanwhile, some studies have observed that in decentralized management modes, conflicts of interest between parent and subsidiary companies may lead to opportunistic behaviors by subsidiaries that the parent company cannot control (Aghion and Tirole, 1997; Aghion et al., 2021) [47,48].

From the external perspective, researchers emphasize the environmental uncertainty faced by enterprise groups. Chenhall (2003), based on contingency theory, proposed that the choice of management control modes is influenced by the level of environmental uncertainty [49]. Building on this, Christie et al. (2003) identified two approaches for selecting management control modes under uncertain conditions: transferring knowledge to those with authority or transferring authority to those with knowledge [50]. Due to the costs of knowledge transfer, decentralized management emerges as the only path to improve management efficiency (Huang et al., 2021) [51]. Liu et al. (2018) argued that under high environmental uncertainty, decentralized management allows subsidiaries to better utilize specialized knowledge, enhancing the enterprise group’s ability to respond to risks [52].

In summary, existing literature on digital transformation and choice of management control modes provides a theoretical foundation for this study but reveals several gaps. First, most research examines the impact of digital transformation on information governance and knowledge transfer in enterprises but fails to explore how enterprise groups choose management control modes under such influence. Second, while research has investigated the mechanisms of management control modes selection from the perspective of balancing knowledge transfer costs and agency costs, few studies focus on how this balance is achieved within enterprise groups (Huang et al., 2017) [53]. Furthermore, empirical evidence addressing these issues from a digital information perspective remains limited (Dobrajska et al., 2015) [54]. To address these gaps, this study takes the wave of digital transformation as its research context, utilizes data from enterprise groups, and positions management control modes selection as the ultimate objective. In addition, this study empirically examines the impact of digital transformation on management control modes selection and verifies the underlying mechanism.

### 2.2. Theoretical analysis and research hypotheses

#### 2.2.1. Digital transformation and the choice of management control modes in enterprise groups.

First, from the perspective of knowledge transfer costs, the advancement of digital transformation provides a theoretical basis for enhancing enterprise knowledge management efficiency (Vial, 2019) [33]. Through artificial intelligence, knowledge management can significantly improve the efficiency of identifying, converting, applying, and innovating proprietary knowledge (Hercheui et al., 2020; Mikalef et al., 2021; Wu et al., 2021; Shan et al., 2021, Li et al., 2024) [55–59], because technical and managerial personnel will use digital technology to filter valuable knowledge and convert it into specific programs, which will be solidified into artificial intelligence systems (Shan et al., 2021) [58]. Moreover, enterprise groups can utilize digital technologies like the internet to acquire proprietary knowledge specific to certain environments, reducing the trial-and-error costs of using such knowledge in different contexts and thereby converting knowledge into benefits (Olszak et al., 2018; Liu et al., 2020; Zhang and Deng, 2024) [60–62]. For the management of subsidiaries directly involved in specific business operations and knowledge accumulation, digital transformation enables the extraction of more discrete and complex knowledge, which can be converted and encoded into big data for use (Wu et al., 2021) [57]. As a result, digital transformation provides top managers with scientific investment strategies, reducing investment biases caused by subjective judgement of top managers (Zhai et al., 2025) [63]. Thus, under the drive of digital technology, the extensive proprietary knowledge of subsidiaries can be disseminated in the form of programs and codes, reducing knowledge transfer costs (Liu et al., 2020) [61]. This reformation allows the parent company to understand and learn the proprietary knowledge necessary for decision-making quickly, facilitating more effective centralized management (Li et al., 2024; Zhang and Deng, 2024) [59,62]. Additionally, enterprise groups enhance cross-regional and cross-departmental communication and collaboration between the parent and subsidiary companies through data-driven, human-machine collaboration methods (Liu et al., 2020; Zhao et al., 2023) [64,65]. Digital technology improves knowledge transfer efficiency and reduces knowledge transfer costs, thereby aiding the parent company in implementing centralized management more efficiently (Vial, 2019) [33]. Thus, according to the theory of knowledge transfer costs, digital transformation reduces the knowledge transfer costs between parent and subsidiary companies, providing the parent company with the proprietary knowledge needed for decision-making, leading enterprise groups to select centralized management control modes.

Second, from the perspective of agency costs, during the implementation of digital transformation, the parent company reshapes the information environment by enhancing its data collection, utilization, and information processing efficiency, extracting valuable information from data sources (Zhu, 2019; Ma and Chen, 2022, Qiao et al., 2024) [66–68]. As a combination of finance and emerging digital technology, digital transformation can effectively circumvent the financial exclusion effect of traditional finance and improve financial services in terms of availability, convenience, and comprehensiveness (Xia and Kong, 2024) [69]. Specifically, after digital transformation, enterprise groups can better understand subsidiaries’ business transactions, financial health, and development prospects using ERP software and big data platforms, effectively reducing information asymmetry between parent and subsidiary companies (Zhang et al., 2021) [70]. Such technical advancement facilitates better supervision and control by the parent company over the subsidiaries, thereby reducing agency costs (Bloom et al., 2014) [26]. Additionally, the parent company can better standardize and encode subsidiaries’ production and operational information using digital technology, making it easier for the parent company to grasp and interpret the internal information of subsidiaries (Pagani, 2013) [71]. The transparency of subsidiaries’ condition enhances internal governance efficiency within the enterprise groups and reduces the supervisory costs of decentralized management (Wu et al., 2021; Liu et al., 2024) [57,72]. Furthermore, as digital transformation progresses, the management control logic enabled by digital technology is embedded in the daily operations of the enterprise groups (Xia and Kong, 2024) [69]. The transparency in production, operations, and financial management processes reduces the supervisory and agency costs of measuring and controlling subsidiary behavior by the parent company (Goldfarb and Tucker, 2019; Daron et al., 2007; Yao, et al., 2023; Chen et al., 2022) [42,73–75]. Thus, digital transformation builds a platform for communication and information exchange between parent and subsidiary companies, breaking down internal information barriers within the enterprise groups (Chapman and Kihn, 2009; Li et al., 2021; Bertani et al., 2021; Zhang et al., 2023) [76–79], thereby reducing the agency costs associated with decentralized management. Therefore, digital transformation enhances the transparency and accessibility of subsidiary data, alleviating principal-agent issues between parent and subsidiary companies and reducing agency costs, making decentralized management control modes more reasonable for enterprise groups. Based on the analysis above, this paper proposes the following hypotheses:

Hypothesis 1a: Digital transformation promotes the choice of centralized management control modes in enterprise groups.

Hypothesis 1b: Digital transformation promotes the choice of decentralized management control modes in enterprise groups.

#### 2.2.2. Digital transformation, internal control quality, the choice of management control modes in enterprise groups.

There is also a causal relationship between the quality of internal control in enterprise groups and the choice of management control modes. Existing literature indicates that improving internal control quality can effectively alleviate principal-agent problems (Patterson and Smith, 2007; Li et al., 2011; Shen et al., 2024) [80–82]. Good information environment can enhance the internal control quality of enterprise groups (Li et al., 2011) [81]. The geographical and institutional differences between parent and subsidiary companies cause information asymmetry (Kang and Kim, 2008; Ghoul et al., 2013) [83,84], posing challenges to the internal control quality from a control environment perspective. Additionally, as external investors, parent companies often struggle to fully understand the internal operations of subsidiaries due to factors such as proprietary knowledge and supervisory distance (Graham et al., 2015; Myers and Majluf, 1984; Robinson and Stocken, 2013) [30,85,86], increasing the difficulty of decentralized management (Melumad and Reichelstein, 1987) [87]. To address this issue, parent companies typically choose centralized management control modes to strengthen control activities and reduce information asymmetry caused by control environments (Graham et al., 2015) [30]. However, digital transformation provides the necessary conditions for improving internal control quality in enterprise groups, enabling them to opt for decentralized management control modes (Qi and Xiao, 2020) [40].

According to the COSO definition of internal control, it is a process implemented by the board of directors and management to provide reasonable assurance regarding the achievement of operational efficiency and effectiveness, and the reliability of financial reporting (Zhang et al., 2017; Yan et al., 2024) [1,88]. Within enterprise groups, management ensures the quality of internal control standards and procedures, constructing a comprehensive and transparent information transmission system, which supports the parent company’s ability to effectively grasp the real internal information of the enterprise group (Bushman and Smith, 2001; Beneish et al., 2008; Feng et al., 2009; Dorantes et al., 2013; Tao et al., 2024) [89–93]. Therefore, exploring the mechanism through which digital transformation affects the choice of management control modes from the perspective of internal control quality in enterprise groups is necessary. First, good internal control quality allows the parent company to monitor the subsidiary’s financial status and operational results in real-time, enhancing the reliability of the subsidiary’s disclosed information and improving the overall transparency of the enterprise groups (Cheng et al., 2024; Dessein, 2002; Zhu, 2004; Doyle et al., 2007a; Doyle et al., 2007b) [25,94–97]. For enterprise groups with high internal control quality, the information disclosed in the subsidiaries’ financial reports is more complete and standardized, accurately reflecting the subsidiary’s internal capital structure and profitability (Altamuro and Beatty, 2010; De Franco et al., 2011; Gallemore and Labro, 2015; Cui and Wang, 2023) [98–101]. When the subsidiary’s behavior and information quality are within controllable limits, enterprise groups are more likely to choose decentralized management control modes (Dessein, 2002) [94]. Second, from the perspectives of the control environment, control activities, information, and communication, digital transformation provides a platform for the effective implementation of internal controls within enterprise groups (Zhang et al., 2023) [79]. Digital transformation includes digitizing and encoding traditional work scenarios, constructing data platforms for real-time data collection, analysis, and visualization, and forming a decentralized, networked, and flattened internal control structure (Qi and Xiao, 2020; Verhoef et al., 2021; Li et al., 2024) [40,102,103]. The transformation process significantly reduces the parent company’s discretionary power over the subsidiary’s daily operations, making traditional top-down central control modes ineffective (Finkelstein and Hambrick, 1990; Liang and Li, 2024) [104,105], and leading to the adoption of decentralized management control modes. Therefore, this paper proposes the following hypothesis:

Hypothesis 2. Digital transformation improves the internal control quality of enterprise groups, leading to the selection of decentralized management control modes.

## 3. Research design

### 3.1. Data

This study selects annual observation data from Chinese A-share listed companies from 2010 to 2022 as the research object. The starting point of 2010 is chosen because the major push for digital transformation in enterprises occurred after this year in China (Yuan et al., 2021; Wu et al., 2022; Huang et al., 2023) [3,106,107]. The initial research sample is processed as follows: (1) Financial industry samples are excluded; (2) ST and * ST companies are excluded; (3) Samples with negative equity are excluded; (4) Samples from IPO years are excluded; (5) Samples with missing data are excluded. It is worth noting that technology firms may have already adopted digital technologies and reported their use in annual reports prior to the broader push for digital transformation. To account for the unique characteristics of the technology sector and to avoid potential sample selection bias, this study excludes observations from technology firms. After applying these filters, the final sample consists of 29,089 firm-year observations.

The management discussion and analysis (MD&A) textual data used in this study are sourced from the Chinese Research Data Service Platform (CNRDS [CNRDS is a specialized data platform providing textual analysis for financial disclosure of Chinese listed firms for professionals. Visit the CNRDS website (https://www.cnrds.com/) for more information]), we collected digital transformation data from listed companies using CNRDS Database. Financial and corporate governance data are primarily obtained from the China Stock Market & Accounting Research Database (CSMAR [CSMAR database is a reliable source for collecting China-listed firm data, and a comprehensive resource widely accessible beyond Chinese academic institutions. CSMAR database extends its reach to over 100 overseas academic institutions, including esteemed universities such as Harvard University, Yale University, University of Chicago, University of Hong Kong, University of Manchester, Monash University, University of Sydney, and others. Its usage extends across scholarly circles, with researchers from institutions like National University of Singapore, Emory University, University of New Hampshire, Nanyang Technological University, and Massey University actively utilizing this database in their academic endeavors. Recognizing that accessing China-specific data sources within CSMAR might pose challenges for some scholars, our commitment to data reliability and transparency remains steadfast. We assure that upon reasonable request, access to the original data supporting our research is readily available. Visit the CSMAR website (https://www.gtarsc.com/) for further details]), the most commonly used academic databases covering Chinese listed firms. It is important to note that this study focuses on the choice of management control modes in enterprise groups, so the sample includes only companies where the controlling shareholder is a corporate entity. To avoid the influence of outliers, all continuous variables are winsorized at the 1% and 99% levels.

### 3.2. Variables

#### 3.2.1. Explained variable: Management control mode.

This study constructs a measurement index for the management control mode from four dimensions: personnel authority, investment authority, operational authority, and financial authority. Utilizing the “dual disclosure system” in the financial information disclosure of Chinese listed companies, we calculate these dimensions and synthesize them into an overall index for the enterprise group’s management control mode (*Mcm*) using principal component analysis (PCA).

**Personnel authority concentration:** Following the method of Pan et al. (2018) [18], the degree of personnel authority concentration is measured by the concentration of employee compensation paid by the enterprise group, reflecting the parent company’s control over personnel decisions within the group. The regression of model (1) is conducted annually and by industry, with the estimated residuals serving as the metric for the concentration of personnel authority. A higher value indicates a greater concentration of personnel authority. In model (1), the dependent variable is the proportion of employee compensation paid by the parent company (*PSalary*), calculated by dividing the “Cash paid to employees” item in the parent company’s cash flow statement by the corresponding item in the consolidated financial statements. The independent variable is the proportion of assets held by the parent company (*PAssets*), calculated by dividing the parent company’s “Total assets” by the “Total assets” in the consolidated financial statements.


$$PSalary_{i,t}\;=\;\beta_{0}\;+\;\beta_{1}PAssets_{i,t}\;+\;\varepsilon_{i,t}$$ (1)

**Investment authority concentration:** Following the method of Tan and Chen (2019) [108], the distribution of investment authority within the group is determined using the “Intangible assets” data from the parent and consolidated financial statements. The regression of model (2) is conducted annually and by industry, with the estimated residuals serving as the metric for the concentration of investment authority. A higher value indicates a greater concentration of investment authority. In model (2), the dependent variable (*PIntangibleAssets*) is the ratio of “Intangible assets” in the parent company’s financial statements to those in the consolidated financial statements; the independent variable (*PAssets*) is the ratio of the parent company’s “Total assets” to the “Total assets” in the consolidated financial statements.


$$PIntangibleAssets_{i,t}\;=\;\beta_{0}\;+\;\beta_{1}PAssets_{i,t}\;+\;\varepsilon_{i,t}$$ (2)

**Financial authority concentration:** Following the method of Zhang and Wu (2011) [109], the concentration of financial authority within the enterprise group is determined using the “Cash” data from the parent and consolidated financial statements. The regression of model (3) is conducted annually and by industry, with the estimated residuals serving as the metric for the concentration of financial authority. A lower value indicates a greater concentration of financial authority. In model (3), the dependent variable (*Cashdis*) equals 1 minus the ratio of cash in the parent company’s statements to cash in the consolidated statements. *Asset* is the natural logarithm of total assets, *Lev* is the debt-to-asset ratio, *P/B* is the price-to-book ratio, *Divsf* is the number of industries involved in the main business, *Assetdis* equals 1 minus the ratio of total assets in the parent company’s statements to total assets in the consolidated statements, *Ocfdis* equals 1 minus the ratio of cash received from sales of goods and services in the parent company’s statements to the consolidated statements, *Div* is a dummy variable for cash dividends (1 for years with dividends, 0 otherwise), and *SEO* is a dummy variable for equity refinancing (1 for years with equity refinancing such as additional issuance or rights issue, 0 otherwise).


$$Cashdis_{i,t}\;=\;\beta_{0}\;+\;\beta_{1}Asset_{i,t}\;+\;\beta_{2}Lev_{i,t}\;+\;\beta_{3}P/B_{i,t}\;+\;\beta_{4}Divsf_{i,t}\;+\;\beta_{5}Assetdis_{i,t}\;+\;\beta_{6}Ocfdis_{i,t}\;+\;\beta_{7}Div_{i,t+1}\;+\;\beta_{8}SEO_{i,t}\;+\;\varepsilon_{i,t}$$ (3)

**Operational authority concentration:** Following the method of Wang and Zhang (2014) [110], the concentration of operational authority is measured by the proportion of operating income from the parent company’s income statement relative to the consolidated financial statements. A higher value indicates a greater concentration of operational authority.

#### 3.2.2. Explanatory variable: Digital transformation.

Drawing from the research by Wu et al. (2021) [4], this study measures the degree of digital transformation (*Digital*) by counting the frequency of the term “digital” in the management discussion and analysis (MD&A) section of annual reports of listed companies. Specifically, this paper extracts the frequencies of terms related to five dimensions: artificial intelligence, big data technology, blockchain technology, cloud computing technology, and the application of digital technology. The sum of these frequencies is then logged to measure the extent of digital transformation in enterprise groups. S1 Appendix lists these keywords, which are adopted to construct a relatively objective and complete digital glossary.

#### 3.2.3. Mediating variable.

The mediating variable in this study is internal control quality. We follow the approach of previous studies (Lu et al., 2015; Cao et al., 2022; Zhang et al., 2023) [111–113] and use the internal control index from the Dibo (The internal control index is sourced from Dib Enterprise Risk Management Technology Co. LTD, which is a composite index that reflects the internal control quality based on the listed firm’s internal control disclosure, internal control assessment, and auditing/assurance reports. Scholars seeking access to relevant data can visit the official website https://www.dibcn.com/to purchase the Internal Control data. While acknowledging potential limitations in accessing China-specific Internal Control data sources, our commitment lies in ensuring the reliability and transparency of the data sources utilized in our study. Should there be reasonable requests, we are prepared to provide access to the original data underpinning our research). Database as a measure of internal control quality (*Icq*) in enterprise groups, calculated as the internal control index divided by 100.

#### 3.2.4. Control variables.

Based on previous research (Yuan et al., 2021; Wang et al., 2014; Chen et al., 2020; Hou and Liu, 2020; Wang et al., 2020) [3,114–117], this study includes the following control variables: firm size (*Size*), leverage ratio (*Lev*), return on total assets (*ROA*), revenue growth rate (*Growth*), board size (*Board*), proportion of independent directors (*Inddir*), CEO duality (*Dual*), ownership concentration (*Top1*), ownership type (*SOE*), firm age (*Age*), and management shareholding ratio (*Manhold*). The definitions and measurements of all variables are provided in Table 1.

**Table 1:** Definitions of variables.

Variables	Symbols	Definitions
Management control mode	Mcm	Constructed using principal component analysis (PCA) from four dimensions: personnel authority, investment authority, operational authority, and financial authority
Digital transformation	Digital	Natural logarithm of the frequency of terms related to digital transformation (including artificial intelligence, big data technology, blockchain technology, cloud computing technology, and digital technology) plus one
Internal control quality	Icq	Dibo internal control index divided by 100
Firm size	Size	Natural logarithm of the company’s total assets at the end of the period
Leverage ratio	Lev	Ratio of the company’s total liabilities to total assets at the end of the period
Return on assets	ROA	Ratio of the company’s net profit to total assets at the end of the period
Revenue growth rate	Growth	(Current main business income - previous period’s main business income)/previous period’s main business income
Board size	Board	Natural logarithm of the total number of board members
Proportion of independent directors	Inddir	Number of independent directors divided by the total number of board members
CEO duality	Dual	Dummy variable, equals 1 if the chairman and CEO positions are held by the same person, otherwise 0
Ownership concentration	Top1	Ratio of shares held by the largest shareholder to the total shares of the company
State ownership	SOE	Dummy variable, equals 1 if the controlling shareholder is a state-owned entity or state-owned legal person, otherwise 0
Firm age	Age	Natural logarithm of the number of years since the company’s IPO
Management shareholding ratio	Manhold	Ratio of shares held by management to the total shares of the company
Year	Year	Dummy variables to control for year-specific effects
Industry	Ind	Dummy variables to control for industry-specific effects

### 3.3. Model construction

To test hypotheses 1a and 1b, the following model is established:


$$Mcm_{i,t}\;=\;\beta_{0}\;+\;\beta_{1}Digital_{i,t}\;+\;\sum Controls\;+\;\sum Ind\;+\;\sum Year\;+\;\varepsilon_{i,t}$$ (4)

In model (4), *Mcm*_*i,t*_ is the management control mode index for company ***i*** in year ***t***, *Digital*_*i,t*_ represents the degree of digital transformation for company ***i*** in year ***t***. *Controls* represents control variables. *Ind* and *Year* are dummy variables representing industry and year fixed effects. *ε*_*i,t*_ is the random error term.

To test hypothesis 2, referring to the approach of Jiang (2022) [118], this study establishes the following mediation effect models to examine the impact of digital transformation on the internal control quality of enterprise groups:


$$Icq_{i,t}\;=\;\beta_{0}\;+\;\beta_{1}Digital_{i,t}\;+\;\sum Controls\;+\;\sum Ind\;+\;\sum Year\;+\;\varepsilon_{i,t}$$ (5)

$$Mcm_{i,t}\;=\;\beta_{0}\;+\;\beta_{1}Digital_{i,t}\;+\;\beta_{1}Icq_{i,t}\;+\;\beta_{1}Digital_{i,t}\;*\;Icq_{i,t}\;+\;\sum Controls\;+\;\sum Ind\;+\;\sum Year\;+\;\varepsilon_{i,t}$$ (6)

Step 1: Use Model (5) to test the relationship between digital transformation and internal control quality. This step evaluates whether digital transformation improves internal control quality in enterprise groups. Step 2: Use Model (6) to examine the relationship among digital transformation, internal control quality, and the management control mode of enterprise groups. This step assesses whether internal control quality mediates the effect of digital transformation on management control modes selection and evaluates the interaction effect.

## 4. Empirical results and analysis

### 4.1. Descriptive statistical analysis

Table 2 presents the descriptive statistics of the variables. According to Table 2, the mean of the management control mode is −0.043 with a standard deviation of 0.988, indicating significant variability in the choice of management control modes among the sample companies. The mean of digital transformation is 1.239, with a median of 1.099, suggesting that the sample companies are generally engaged in exploring digital transformation. The minimum value is 0, and the maximum value is 5.063, indicating substantial differences in the levels of digital transformation among different companies. The mean of internal control quality is 6.405, with a median of 6.648, suggesting minimal differences in internal control quality among the sample companies. The distribution of values for other control variables aligns with the actual conditions of listed companies, and no anomalies were found.

**Table 2:** Results for descriptive statistics.

Variable	N	Mean	Median	SD	Min	Max
Mcm	29089	−0.043	−0.045	0.988	−1.768	1.721
Digital	29089	1.239	1.099	1.261	0	5.063
Icq	28998	6.405	6.648	1.267	0	8.385
Size	29089	22.260	22.070	1.272	19.980	26.210
Lev	29089	0.431	0.425	0.202	0.057	0.882
ROA	29089	0.056	0.053	0.066	−0.202	0.258
Growth	29089	0.164	0.107	0.366	−0.536	2.100
Board	29089	2.126	2.197	0.197	1.609	2.708
Inddir	29089	0.376	0.357	0.054	0.333	0.571
Dual	29089	0.280	0	0.449	0	1
Top1	29089	0.344	0.322	0.145	0.091	0.738
SOE	29089	0.350	0	0.477	0	1
Age	29089	2.144	2.303	0.776	0.693	3.332
Manhold	29089	0.134	0.006	0.194	0	0.677

### 4.2. Correlation analysis

Table 3 presents the correlation analysis results of the main variables. As shown in Table 3, the coefficient between digital transformation (*Digital*) and the management control mode (*Mcm*) of enterprise groups is significantly negative (−0.102, p < 0.01), consistent with the direction predicted by hypothesis 1b. This indicates that digital transformation significantly reduces the degree of centralization in enterprise groups. The coefficients of *Icq* (internal control quality) with *Digital* and *Mcm* are both insignificant (−0.005, p > 0.1; −0.007, p > 0.1), suggesting that the mediating effect of internal control quality between digital transformation and the management control mode requires further testing. Moreover, the correlation coefficients between explanatory and control variables are all less than 0.5, and the variance inflation factor (VIF) values in the regression analysis are below 10, indicating no severe multicollinearity issues among the variables.

**Table 3:** Correlation matrix.

Variable	Mcm	Digital	Icq	SOE	Size	Lev	ROA	Growth	Board	Inddir	Dual	Top1	Age	Manhold
Mcm	1													
Digital	−0.102***	1												
Icq	−0.005	−0.007	1											
SOE	−0.129***	−0.131***	0.044***	1										
Size	−0.428***	0.084***	0.123***	0.351***	1									
Lev	−0.328***	−0.023***	−0.083***	0.271***	0.500***	1								
ROA	0.035***	−0.041***	0.373***	−0.058***	0.058***	−0.276***	1							
Growth	−0.085***	0	0.180***	−0.062***	0.047***	0.032***	0.314***	1						
Board	−0.051***	−0.094***	0.053***	0.282***	0.254***	0.145***	0.047***	−0.002	1					
Inddir	−0.006	0.068***	−0.003	−0.062***	0	−0.005	−0.033***	−0.003	−0.545***	1				
Dual	0.086***	0.091***	−0.004	−0.302***	−0.180***	−0.124***	0.007	0.024***	−0.195***	0.116***	1			
Top1	0.023***	−0.068***	0.127***	0.198***	0.186***	0.024***	0.136***	0.008	0.016***	0.050***	−0.045***	1		
Age	−0.372***	−0.008	−0.093***	0.461***	0.416***	0.354***	−0.141***	−0.087***	0.164***	−0.029***	−0.255***	−0.085***	1	
Manhold	0.229***	0.078***	0.041***	−0.479***	−0.333***	−0.298***	0.106***	0.060***	−0.206***	−0.029***	0.252***	−0.084***	−0.556***	1

***Significant at 1%; **Significant at 5%; *Significant at 10%.

### 4.3. Baseline regression results

Table 4 presents the results of the multiple regression analysis on the impact of digital transformation on the choice of management control modes in enterprise groups. The results in columns (1) and (2) show a significant negative correlation at 1% level between digital transformation and the choice of management control modes, regardless of whether other factors are controlled for (−0.082, p < 0.01; −0.032, p < 0.01). This indicates that the higher the level of digital transformation in enterprise groups, the more decentralized the corresponding attributes in their management control modes, leading to the selection of decentralized management control modes. Thus, hypothesis 1b is tested.

**Table 4:** Results for multivariate regression analysis using OLS method.

	(1)	(2)
Variables	Mcm	Mcm
Digital	−0.082***	−0.032***
	(−15.54)	(−6.76)
SOE		0.204***
		(15.38)
Size		−0.260***
		(−48.51)
Lev		−0.505***
		(−15.81)
ROA		0.301***
		(3.49)
Growth		−0.246***
		(−17.26)
Board		0.341***
		(10.67)
Inddir		0.006***
		(5.35)
Dual		−0.014
		(−1.22)
Top1		0.002***
		(5.63)
Age		−0.286***
		(−32.99)
Manhold		0.001***
		(3.22)
Constant	0.058***	5.542***
	(6.86)	(42.67)
Ind	Yes	Yes
Year	Yes	Yes
Observations	29089	29089
R-squared	0.124	0.322
Adj_R^2^	0.123	0.321

*t* statistics are in parentheses.

*p < 0.1, **p < 0.05, ***p < 0.01.

### 4.4. Mechanism test

To test hypothesis 2, this study examines the mechanism through which digital transformation influences the choice of management control modes in enterprise groups, building on the verification of hypothesis 1b and following the approach of Jiang (2022) [118]. The regression results are presented in Table 5.

**Table 5:** Regression results for mechanism test.

	(1)	(2)
Variables	Icq	Mcm
Digital	0.027***	−0.114***
	(4.14)	(−5.70)
Icq		−0.018***
		(−3.17)
Digital_Icq		0.013***
		(4.20)
SOE	0.198***	0.201***
	(10.72)	(15.12)
Size	0.189***	−0.259***
	(25.31)	(−47.66)
Lev	−0.561***	−0.510***
	(−12.59)	(−15.91)
ROA	5.756***	0.284***
	(48.38)	(3.20)
Growth	0.241***	−0.246***
	(12.11)	(−17.17)
Board	0.006	0.344***
	(0.14)	(10.75)
Inddir	0.002	0.006***
	(1.13)	(5.45)
Dual	0.035**	−0.014
	(2.17)	(−1.19)
Top1	0.003***	0.002***
	(5.56)	(5.58)
Age	−0.134***	−0.286***
	(−11.08)	(−32.87)
Manhold	0.002***	0.001***
	(4.38)	(3.13)
Constant	2.041***	5.621***
	(11.23)	(42.39)
Ind	Yes	Yes
Year	Yes	Yes
Observations	28,998	28,998
R-squared	0.200	0.321
Adj_R^2^	0.199	0.320

*t* statistics are in parentheses.

*p < 0.1, **p < 0.05, ***p < 0.01.

As shown in Table 5, the regression in model (5) indicates that the regression coefficient of digital transformation in column (1) is significantly positive at the 1% level (0.027, p < 0.01). This implies that digital transformation significantly enhances the quality of internal control within enterprise groups. In column (2), the regression coefficient for internal control quality in Model (6) is significantly negative (−0.018, p < 0.01), suggesting that higher internal control quality leads to more decentralized management control modes in enterprise groups. These results demonstrate that digital transformation enhances the internal control quality of enterprise groups, facilitating communication between parent and subsidiary companies and thereby promoting the adoption of decentralized management control modes. Thus, hypothesis 2 is tested.

## 5. Robustness tests

### 5.1. Replacement of the explained variable

Following the methodology of Pan et al. (2018) [18], to control for the potential impact of executive compensation on the calculation of the personnel authority index in enterprise groups, this study excludes executive compensation from both the numerator and denominator of the *PSalary* variable. Specifically, when the proportion of executives in the parent company is high and their compensation is substantial, most of the compensation paid by the parent company constitutes executive compensation. This phenomenon will lead to higher calculated concentrations of personnel authority even when a decentralized management control mode is chosen, thereby causing noise. Therefore, this study excludes executive compensation from the *PSalary* calculation formula and recalculates the personnel authority concentration index according to model (1). Subsequently, the management control mode choice indicator *Mcm* is recomposed. The regression results remain robust (−0.060, p < 0.01; −0.035, p < 0.01), as shown in Table 6.

**Table 6:** Robust test results for replacing explained variables.

	(1)	(2)
Variables	Mcm	Mcm
Digital	−0.060***	−0.035***
	(−11.45)	(−7.45)
SOE		0.208***
		(14.72)
Size		−0.242***
		(−42.17)
Lev		−0.557***
		(−16.28)
ROA		0.118
		(1.30)
Growth		−0.262***
		(−16.81)
Board		0.347***
		(10.09)
Inddir		0.006***
		(4.99)
Dual		−0.025**
		(−2.02)
Top1		0.003***
		(8.32)
Age		−0.275***
		(−29.64)
Manhold		0.001***
		(4.07)
Constant	0.061***	5.137***
	(6.97)	(37.06)
Ind	Yes	Yes
Year	Yes	Yes
Observations	26,690	26,690
R-squared	0.093	0.287
Adj_R^2^	0.0914	0.286

*t* statistics are in parentheses.

*p < 0.1, **p < 0.05, ***p < 0.01.

### 5.2. Replacement of the explanatory variable

Following the methodology of Zhao et al. (2021) [119], this study redefines the extent of digital transformation in enterprise groups from four dimensions: the application of digital technology, internet business models, intelligent manufacturing, and modern information systems. We statistically analyze the frequency of the term “digitalization” in the management discussion and analysis sections of listed companies’ annual reports. Based on this frequency data, we standardize the data and use the entropy method to determine the weights of each indicator, ultimately deriving the digital transformation index (*Digital*). After replacing the indicator and excluding samples with missing data, the sample size is reduced to 11,281. However, the regression results remain consistent (−1.774, p < 0.01; −1.474, p < 0.01), as shown in Table 7.

**Table 7:** Robust test results for replacing explanatory variables.

	(1)	(2)
Variables	Mcm	Mcm
Digital	−1.774***	−1.474***
	(−6.17)	(−5.50)
SOE		0.222***
		(10.91)
Size		−0.208***
		(−25.17)
Lev		−0.581***
		(−10.81)
ROA		−0.798***
		(−4.99)
Growth		−0.177***
		(−8.44)
Board		0.371***
		(7.22)
Inddir		0.007***
		(3.98)
Dual		0.072***
		(3.05)
Top1		0.001
		(1.32)
Age		−0.526***
		(−16.67)
Manhold		0.001
		(0.59)
Constant	−0.144***	5.137***
	(−5.80)	(23.10)
Ind	Yes	Yes
Year	Yes	Yes
Observations	11,281	11,281
R-squared	0.147	0.265
Adj_R^2^	0.143	0.261

*t* statistics are in parentheses.

*p < 0.1, **p < 0.05, ***p < 0.01.

### 5.3. Instrumental variable method

To address potential endogeneity issues in model (4), this study adopts the instrumental variable (IV) method. Drawing from research of Huang et al. (2019) [120], we initially select the 1984 postal and telecommunications data of various cities as the instrumental variable for the digitalization of enterprise groups. The communication methods used historically in the enterprises’ locations affect the application and acceptance of information technology during the sample period through aspects such as technological level and social preferences, meeting the relevance condition. Simultaneously, postal and telecommunications services, as part of the social infrastructure, primarily provide communication services to the public and do not directly influence the choice of management control modes in enterprise groups, meeting the exogeneity condition. Furthermore, considering that the 1984 postal and telecommunications data are cross-sectional and cannot be directly used as panel data instrumental variable, we follow Zhao et al. (2020) [121] by using the interaction term between the national internet user count lagged by one period and the number of landline telephones per ten thousand people in each city in 1984 (*Telephone*) as the instrumental variable for the current period’s digital transformation extent.

Columns (1) and (2) of Table 8 present the first-stage regression results for the instrumental variable (*Telephone*). The regression coefficient for *Telephone* is significantly positive at the 1% level (0.000, p < 0.01; 0.000, p < 0.01), indicating a significant positive relationship between the historical penetration of landline telephones and the digital transformation of local enterprise groups, which meets the relevance requirement for the instrumental variable. Columns (3) and (4) of Table 8 report the second-stage regression results of the IV method. The Kleibergen-Paap rk LM statistic is significant at the 1% level (p < 0.01), rejecting the null hypothesis of under-identification of the IV method. The Cragg-Donald Wald F statistic exceeds the critical value for the Stock-Yogo weak instrument test at the 10% significance level, rejecting the null hypothesis of weak instruments. Therefore, the selected instrumental variable is reasonable and reliable. The regression results in Table 8 show that the coefficient of *Digital* is significantly negative (−1.339, p < 0.01; −1.381, p < 0.01), indicating that the main conclusions of this study remain valid.

**Table 8:** Robust test results for IV method.

	(1)	(2)	(3)	(4)
Variables	Digital	Digital	Mcm	Mcm
Telephone	0.000***	0.000***		
	(3.94)	(3.24)		
Digital			−1.339***	−1.381***
			(−3.30)	(−2.80)
Controls	No	Yes	No	Yes
Ind	Yes	Yes	Yes	Yes
Year	Yes	Yes	Yes	Yes
Observations	24815	24815	24815	24815
Kleibergen-Paap rk LM statistic	15.55***	10.52***	15.55***	10.52***
Cragg-Donald Wald F statistic	84.28	56.53	84.28	56.53
	[16.38]	[16.38]	[16.38]	[16.38]

The critical value of the Stock Logo weak instrumental variable identification F-test at the 10% significance level is indicated in square brackets.

### 5.4. Exogenous shock test

Drawing from the methodology of Zhao et al. (2020) [121], this study further utilizes the “Broadband China” policy pilot as an exogenous shock to examine the relationship between digital transformation and the choice of management control modes in enterprise groups. The “Broadband China” policy pilot refers to the selection of 120 demonstration cities (or city clusters) by the Ministry of Industry and Information Technology and the National Development and Reform Commission in 2014, 2015, and 2016, following the “Notice of the State Council on Issuing the ‘Broadband China’ Strategy and Implementation Plan”. The aim is to significantly improve local broadband levels through the construction of demonstration zones, thereby playing a demonstrative and leading role nationwide. This study posits that the construction of “Broadband China” demonstration cities can effectively enhance local broadband infrastructure, providing network support for enterprise digital transformation. Therefore, the following multi-period Difference-in-Differences (DID) model is constructed to test the impact of the “Broadband China” policy pilot on the choice of management control modes in enterprise groups:


$$Mcm_{i,t}\;=\;\beta_{0}\;+\;\beta_{1}TreatPost_{i,t}\;+\;\sum Controls\;+\;\sum Ind\;+\;\sum Year\;+\;\varepsilon_{i,t}$$ (7)

In model (7), *TreatPost* is a dummy variable indicating whether the city of the listed company is included in the “Broadband China” pilot city list for that year (1 if included, 0 otherwise). Other variables are consistent with those in model (1). Table 9 presents the results of the exogenous shock test based on the “Broadband China” policy. The coefficient of *TreatPost* is significantly negative at the 1% level (−0.117, p < 0.01; −0.115, p < 0.01), indicating that the construction of “Broadband China” pilot cities can promote the adoption of decentralized management control modes in enterprise groups. The exogenous shock test based on the “Broadband China” policy supports the conclusion that digital transformation promotes the choice of decentralized management control modes in enterprise groups to some extent.

**Table 9:** Robust test results for exogenous shock testing.

	(1)	(2)
Variables	Mcm	Mcm
Treat_Post	−0.117***	−0.115***
	(−3.52)	(−3.82)
Controls	No	Yes
Ind	Yes	Yes
Year	Yes	Yes
Observations	27,220	27,220
R-squared	0.125	0.329
Adj_R^2^	0.123	0.328

*t* statistics are in parentheses.

*p < 0.1, **p < 0.05, ***p < 0.01.

## 6. Moderating effect tests

### 6.1. Ownership

The number of state-owned enterprises (SOEs) in China is substantial, and there are noteworthy distinctions between SOEs and non-SOEs (Wu et al., 2023) [122]. For different types of enterprise groups, state-owned and non-state-owned enterprise groups face varying operating environments (Milliken, 1987; George, 2005; Fan et al., 2013; Fitza and Tihanyi, 2017) [123–126]. Consequently, the allocation of powers such as personnel, financial, operational, and investment authorities may differ between state-owned enterprise (SOE) groups and non-SOE groups, leading to different selections of management control modes (Pan et al., 2018, Huang et al., 2017) [18,53]. From the perspective of the internal process of “receiving, identifying, and feedback” of information, SOE groups can use digital transformation to build information platforms, which not only improve the quality of generated information but also promote the rapid transmission of subsidiary information to the parent company (Liu et al., 2024) [2]. Since SOE groups are subject to strict supervision by the State-owned Assets Supervision and Administration Commission and are equipped with rigorous performance evaluation systems, they tend to uniformly manage and control subsidiary information to avoid the concealment of financial conditions and operational risks by subsidiary management (Luo, 2012) [127]. In the context of digital transformation, SOE groups enhance their corporate governance internally, reduce agency costs between parent and subsidiary companies, and alleviate principal-agent issues (Liu et al., 2020, Tao et al., 2024) [61,93], making it more favorable for the parent company to choose decentralized management control modes. Therefore, this study posits that the effect of digital transformation in promoting the choice of decentralized management control modes is more pronounced in SOE groups.

To test the moderating effect of ownership on the relationship between digital transformation and the choice of management control modes in enterprise groups, this study constructs a dummy variable for ownership (*SOE*), which takes the value of 1 for SOE groups and 0 otherwise. Column (1) of Table 10 shows the regression results of the moderating effect of ownership. It can be seen that the coefficient of the interaction term between digital transformation and ownership (*Digital_SOE*) is significantly negative at the 1% level (−0.065, p < 0.01), indicating that the role of digital transformation in promoting the choice of decentralized management control modes is more evident in SOE groups.

**Table 10:** Regression results for moderating effects.

	(1)	(2)	(3)
Variables	Mcm	Mcm	Mcm
Digital	−0.014***	−0.031***	−0.036***
	(−2.60)	(−3.98)	(−7.53)
SOE	0.278***		
	(16.88)		
Digital_SOE	−0.065***		
	(−7.56)		
EU		−0.089***	
		(−11.23)	
Digital_EU		−0.009**	
		(−2.20)	
Gov			−0.196***
			(−9.91)
Digital_Gov			0.017***
			(4.41)
Controls	Yes	Yes	Yes
Ind	Yes	Yes	Yes
Year	Yes	Yes	Yes
Observations	29,089	22,215	28,417
R-squared	0.324	0.279	0.322
Adj_R^2^	0.322	0.277	0.321

*t* statistics are in parentheses.

*p < 0.1, **p < 0.05, ***p < 0.01.

### 6.2. Environmental uncertainty

The high uncertainty of the market environment has become a norm in the new economy (Yang et al., 2022) [128]. Increased environmental uncertainty implies higher operational risks for enterprise groups (Huber et al., 1975) [129], significantly impacting their organizational structures and decision-making behaviors (De Sarbo et al., 2005; Ghosh and Olsen, 2009) [130,131], and consequently influencing their choice of management control modes. The aim of digital transformation is to respond promptly to uncertain environmental events, converting uncertainty into certainty, and thus the management control modes choice of enterprise groups will adjust according to changes in external environmental uncertainty (Zhao et al., 2020) [121]. Specifically, digital transformation can enhance decision-making efficiency and organizational resilience in enterprise groups (Qi et al., 2021; Bloom et al., 2014) [20,26]. Utilizing big data and cloud collaboration platforms, parent and subsidiary companies can make real-time decisions and collaborate in exploring opportunities (George et al., 2014; Troilo et al., 2017; Feng et al., 2024) [132–134]. At the strategic level, digitalization encourages subsidiaries to actively seek innovation, optimize resource allocation, and rearrange power structures, turning “crisis” into “opportunity” in highly uncertain environments (Shan et al., 2021, Feng et al., 2024) [58,134]. Therefore, enterprise groups with higher levels of digital transformation exhibit greater resilience in uncertain environments, reducing the pressure on subsidiary management and the motivation to engage in opportunistic behavior (Guo et al., 2023) [135]. By alleviating principal-agent problems, enterprise groups are more inclined to choose decentralized management control modes (Jensen and Meckling, 1992) [19]. Thus, this study posits that the effect of digital transformation in promoting the choice of decentralized management control modes is more obvious when enterprise groups face higher environmental uncertainty.

To test the moderating effect of environmental uncertainty on the relationship between digital transformation and the choice of management control modes in enterprise groups, this study follows the approach of Shen et al. (2012) [136]. We measure sales revenue volatility over the past five years, excluding growth and industry factors, and construct model (8) to estimate the abnormal sales revenue of enterprises over the past five years, excluding growth factors. In model (8), *Year* is a sequence variable taking values from 5 to 1 for years t to (t-4), and *ε* is the residual term representing abnormal sales revenue excluding growth factors. The standard deviation of the residuals (*ε*) over the past five years, adjusted by industry and annual medians, divided by its mean value, is used as the environmental uncertainty indicator (*EU*). A higher *EU* value indicates greater environmental uncertainty.


$$Sale\;=\;\beta_{0}\;+\;\beta_{1}Year\;+\;\varepsilon$$ (8)

Column (2) of Table 10 presents the regression results of the moderating effect of environmental uncertainty. It shows that the coefficient of the interaction term between digital transformation and environmental uncertainty (*Digital_EU*) is significantly negative at the 1% level (−0.009, p < 0.01), indicating that in highly uncertain environments, the advantages of digital transformation in stabilizing operations and turning “crisis” into “opportunity” are more pronounced, and enterprise groups are more inclined to choose decentralized management control modes in uncertain environments.

### 6.3. Corporate governance level

There is a significant causal relationship between the choice of management control modes in enterprise groups and their internal governance levels (Yuan et al., 2021) [3]. Enterprise groups with imperfect internal governance often exhibit internal control deficiencies (Shan, 2010) [137] and frequent board meetings (Cai and Wu, 2007) [138], indicating more internal risks and a tendency to choose centralized management control modes. Digital transformation can enhance the information processing capabilities of enterprise groups, improve the accuracy and timeliness of information disclosure, help parent companies identify and rectify issues, and thereby improve corporate governance levels (Sui et al., 2024, Bertani et al., 2021) [34,78]. Good internal governance environment within enterprise groups can better supervise and constrain the production and operation behaviors of subsidiaries (Long et al., 2011; Hazarika et al., 2012) [139,140], restrict inappropriate behaviors such as seeking on-the-job consumption and building “corporate empires”, align the goals of parent and subsidiary companies, and reduce principal-agent problems (Shen et al., 2024, Bushman and Smith, 2001) [82,89], thereby creating conditions for the choice of decentralized management control modes. Therefore, from the perspective of principal-agent theory, digital transformation and corporate governance exert equivalent regulatory effects, prompting enterprise groups to choose decentralized management control modes.

To test the moderating effect of corporate governance level on the relationship between digital transformation and the choice of management control modes in enterprise groups, this study follows the approach of Zhou et al. (2020) [141]. We use principal component analysis (PCA) to construct a comprehensive indicator measuring corporate governance level from various aspects, including supervision, incentives, and decision-making. The incentive mechanism is represented by executive compensation and executive shareholding ratio, board supervision is represented by the proportion of independent directors and board size, and equity structure supervision is represented by the institutional shareholding ratio and equity balance degree. The decision-making power of the general manager is represented by whether the chairman and general manager roles are combined. Based on these seven indicators, we construct a corporate governance index (*Gov*) using PCA, with the first principal component from PCA representing the overall level of corporate governance. A higher value of this index indicates better corporate governance.

Column (3) of Table 10 presents the regression results of the moderating effect of corporate governance level. The results show that the coefficient of the interaction term between digital transformation and corporate governance level (*Digital_Gov*) is significantly positive at the 1% level (0.017, p < 0.01), while the regression coefficients of both digital transformation (*Digital*) and corporate governance level (*Gov*) are significantly negative at the 1% level (−0.036, p < 0.01; −0.196, p < 0.01). This indicates that digital transformation and corporate governance can exert equivalent substitution effects, in other words, the higher the degree of digital transformation or the higher the level of corporate governance, the more likely enterprise groups are inclined to choose decentralized management control modes.

## 7. Conclusion and discussion

### 7.1. Conclusion

To explore the impact of digital transformation on the choice of management control modes in enterprise groups, this study uses data from A-share listed companies from 2010 to 2022. By combining four dimensions: personnel authority, operational authority, investment authority, and financial authority, we construct an indicator of management control mode for enterprise groups and empirically test the relationship between digital transformation and the choice of management control modes, along with its influencing mechanism. The main conclusions are as follows:

1. Promotion of decentralized management control: The advancement of digital transformation promotes the choice of decentralized management control modes in enterprise groups. This conclusion remains robust after various tests for endogeneity and robustness.
2. Mechanism of influence: The mechanism test reveals that digital transformation alleviates principal-agent problems between parent and subsidiary companies by enhancing internal control quality, thereby creating a favorable internal governance environment for the choice of decentralized management control modes.
3. Moderating effects: The moderating effect tests show that the promotion of decentralized management control modes by digital transformation is more pronounced in state-owned enterprise groups and those operating in environments with higher uncertainty. Additionally, the higher the degree of digital transformation or the better the level of corporate governance, the more likely enterprise groups are to choose decentralized management control modes.

### 7.2. Discussion

This study adopts the classic management control theory framework by Jensen and Meckling (1995) [22], using knowledge transfer costs and agency costs as perspectives to construct a research framework for understanding how digital transformation impacts the choice of management control modes in enterprise groups. Existing literature rarely investigates how enterprise groups balance knowledge transfer costs and agency costs during the process of selecting management control modes. In an innovative approach, this study integrates digital transformation into the process of management control modes selection for enterprise groups. The results demonstrate that the information governance effects brought by digital transformation encourage enterprise groups to adopt decentralized management control modes. However, digital transformation also reduces knowledge transfer costs between parent and subsidiary companies, aiding parent companies in acquiring proprietary knowledge. At the same time, it enables parent companies to more effectively identify irrational decisions made by subsidiaries, further facilitating decentralized management. This equilibrium between knowledge transfer costs and agency costs is a crucial new finding, significantly enriching the literature on management control in enterprise groups.

Additionally, this study provides further clarity on the relationships between digital transformation, internal control quality, and management control modes in enterprise groups. Existing research has shown that high-quality internal control can effectively mitigate principal-agent problems (Patterson and Smith, 2007; Li et al., 2011; Shen et al., 2024) [80–82], providing a favorable informational environment for management control in enterprise groups (Beneish et al., 2008; Dorantes et al., 2013) [90,92]. Previous studies have examined the impact of digital transformation on internal control quality (Cheng et al., 2024) [25] and analyzed the relationship between internal control quality and management control modes in enterprise groups (Zhang et al., 2017) [1]. This study makes a pioneering contribution by using internal control quality as a mediating variable in understanding how digital transformation influences the choice of management control modes in enterprise groups. It finds that enterprise groups achieve decentralized management control by improving internal control quality through digital transformation. This finding not only enriches the literature on internal control but also provides a theoretical basis for understanding how enterprise groups can leverage digital transformation in their management control modes selection process.

### 7.3. Implications

The main content and research conclusions of this study have the following implications:

1. Integration of management control and digital technology: In the digital economy era, enterprise groups should integrate management control modes with digital technologies while emphasizing the internal distribution of authority. Digital transformation reduces internal knowledge transfer and agency costs, leading to an evolution in the internal power distribution of enterprise groups. With the trend of decentralizing power to subsidiaries driven by digital transformation, parent companies should further promote the application of digital technologies, establish regulatory defenses within subsidiaries, and preemptively mitigate opportunistic behaviors of management.
2. Internal mechanism of digital transformation: Enterprise groups should focus on the internal mechanism of digital transformation and correctly identify the application areas of digital technologies to increase the success rate of digital transformation. At the micro level, enterprise groups should not blindly invest in digital equipment and technologies but should emphasize how digital technologies can exert their advantages. It is crucial to recognize the role of digital transformation in enhancing internal control quality, thereby improving the application capability of digital technologies within enterprise groups. By enhancing the complementarity between digital and economic entity, enterprise groups can promote the deep integration of digital and entity domains.
3. Adaptation to digital transformation: Enterprise groups that are slow to adjust nowadays should actively use digital transformation to drive internal power restructuring and enhance their adaptability. Since the impact of digital transformation varies among different types of enterprise groups, SOEs and those with higher levels of corporate governance can rely on digital transformation to complete power structure adjustments and adapt to environmental uncertainties. Therefore, enterprise groups that are slow to adjust their management control modes under the impact of digital transformation should accurately use digital technologies to improve supervision and management systems based on information and data, achieving reasonable internal division of labor and authorization.

## Supporting information

S1 Dataset The data set used in this article for discussion and analysis. (ZIP)

S1 Appendix Keywords related to digital transformation. (DOCX)
