# Supplementary material for: Digital transformation and the choice of management control modes in enterprise groups
Source: PLoS One. 2025 Apr 22;20(4):e0320328. doi: 10.1371/journal.pone.0320328 (PMC12013941; doi:10.1371/journal.pone.0320328)
Supplement: S1 Appendix — (DOCX) [file pone.0320328.s002.docx]

| **Appendix** **1.** **Word frequency of digital transformation.** | |
| --- | --- |
| Artificial Intelligence | Artificial Intelligence, Image Understanding, Investment Decision Aids, Intelligent Data Analytics, Intelligent Robots, Machine Learning, Deep Learning, Biometrics, Face Recognition, Speech Recognition, Identity Verification, Autonomous Driving, Natural Language Processing, AI Reasoning, AI Chips, Cancer Detection, Emoji Analysis, Gait Technology, Dialogue Robots, Adversarial Networks, Dialect Translation, Assisted Diagnosis, Computer Vision, Human-Computer Interaction, Neural Networks, Sound Recognition, Visual Tracking, Drones, Unmanned Vehicles, Virtual Reality, Human-Computer Interaction |
| Big Data | Big Data, Data Mining, Text Mining, Data Visualization, Big Data Portrait, Big Data Recommendation, High Frequency Data, Massive Query, Mass Storage, Massive Data, Genetic Information, Transaction Data, Quantitative Finance, Data Acquisition, Data Warehousing, Data Storage, Data Analysis, Data Correlation, Data Management, Data Cleaning, Intelligent Data Analysis, Heterogeneous Data, Augmented Reality, Mixed Reality Data Storage Services, Precision Marketing, Crawlers, Remote Sensing Data, Public Opinion, Data Platforms, Digital Terminals, Digital Intelligence |
| Blockchain | Blockchain, Digital Currency, Distributed Computing, Differential Privacy Technology, Hash Algorithms, Hash Functions, Merkle Trees, Copyright Verification, Bitcoin, Genesis Blocks, Cryptographic Keys, Asymmetric Encryption, Digitization of Contracts, Federated Chains, Decentralization, Digital Signatures, Private Chains, Ether, Smart Contracts |
| Cloud Computing | Cloud Computing, Stream Computing, Graph Computing, Memory Computing, Multi-Party Secure Computing, Brain-Like Computing, Green Computing, Cognitive Computing, Converged Architecture, Information Physical Systems, AliCloud, Edge Computing, Elastic Computing, Distributed Computing, Load Balancing, Network Computing, Virtualization, Virtual Desktops, Cloud Security, Cloud Backup, Cloud Systems, Cloud Services, Cloud Platforms, Cloud Applications, Cloud Games, IaaS, PaaS IaaS, SaaS, Billion Level Concurrency |
| Application of Digital Technology | Mobile Internet, Industrial Internet, Mobile Interconnection, Internet Healthcare, Digital-Twin, E-Commerce, Mobile Payment, Third-Party Payment, NFC Payment, Smart Energy, B2B, B2C, C2B, C2C, O2O, Networking, Smart Wear, Smart Agriculture, Smart Transportation, Smart Healthcare, Smart Customer Service, Smart Home, Smart Investment, Smart Travel, Smart Environmental Protection, Smart Grid, Smart Marketing, Unmanned Retail, Internet Finance, Digital Finance, Fintech, Quantitative Finance, Open Banking |
